# Anaerobic oxidation of short-chain alkanes in hydrothermal sediments: potential influences on sulfur cycling and microbial diversity

**DOI:** 10.3389/fmicb.2013.00110

**Published:** 2013-05-14

**Authors:** Melissa M. Adams, Adrienne L. Hoarfrost, Arpita Bose, Samantha B. Joye, Peter R. Girguis

**Affiliations:** ^1^Department of Organismic and Evolutionary Biology, Harvard UniversityCambridge, MA, USA; ^2^Department of Marine Sciences, University of North Carolina at Chapel HillChapel Hill, NC, USA; ^3^Department of Marine Sciences, University of GeorgiaAthens, GA, USA

**Keywords:** hydrothermal vent, metalliferous sediments, Juan de Fuca Ridge, short-chain alkanes, sulfate reduction

## Abstract

Short-chain alkanes play a substantial role in carbon and sulfur cycling at hydrocarbon-rich environments globally, yet few studies have examined the metabolism of ethane (C_2_), propane (C_3_), and butane (C_4_) in anoxic sediments in contrast to methane (C_1_). In hydrothermal vent systems, short-chain alkanes are formed over relatively short geological time scales via thermogenic processes and often exist at high concentrations. The sediment-covered hydrothermal vent systems at Middle Valley (MV, Juan de Fuca Ridge) are an ideal site for investigating the anaerobic oxidation of C_1_–C_4_ alkanes, given the elevated temperatures and dissolved hydrocarbon species characteristic of these metalliferous sediments. We examined whether MV microbial communities oxidized C_1_–C_4_ alkanes under mesophilic to thermophilic sulfate-reducing conditions. Here we present data from discrete temperature (25, 55, and 75°C) anaerobic batch reactor incubations of MV sediments supplemented with individual alkanes. Co-registered alkane consumption and sulfate reduction (SR) measurements provide clear evidence for C_1_–C_4_ alkane oxidation linked to SR over time and across temperatures. In these anaerobic batch reactor sediments, 16S ribosomal RNA pyrosequencing revealed that *Deltaproteobacteria*, particularly a novel sulfate-reducing lineage, were the likely phylotypes mediating the oxidation of C_2_–C_4_ alkanes. Maximum C_1_–C_4_ alkane oxidation rates occurred at 55°C, which reflects the mid-core sediment temperature profile and corroborates previous studies of rate maxima for the anaerobic oxidation of methane (AOM). Of the alkanes investigated, C_3_ was oxidized at the highest rate over time, then C_4_, C_2_, and C_1_, respectively. The implications of these results are discussed with respect to the potential competition between the anaerobic oxidation of C_2_–C_4_alkanes with AOM for available oxidants and the influence on the fate of C_1_ derived from these hydrothermal systems.

## INTRODUCTION

Hydrocarbon gases, including methane (C_1_), ethane (C_2_), propane (C_3_), and* n*-butane (C_4_), are produced via thermogenic and biogenic processes in the deep subsurface and are substantial components of the organic carbon pool across marine and terrestrial ecosystems (Joye et al., 2004; Milkov, 2005; Cruse and Seewald, 2006; Hinrichs et al., 2006; Savage et al., 2010). Over the past decade, studies focused on the anaerobic oxidation of methane (AOM) revealed the functional potential, ecological physiology, and diversity of microorganisms mediating this process and the global distribution of AOM as an effective benthic filter that reduces methane emissions into the oceans and atmosphere (for reviews, see Conrad, 2009; Knittel and Boetius, 2009; Valentine, 2011). In contrast, the anaerobic oxidation of long-chain alkanes (>C_6_) and aromatics has also been studied extensively resulting in the isolation of several bacteria, such as sulfate-reducing bacteria (SRB) that oxidize crude oil anaerobically (Van Hamme et al., 2003). There is a gap in our understanding of the metabolism and fate of non-methane, short-chain (C_2_–C_4_) alkanes in deep sea sediments. Furthermore, there is growing interest in determining the extent to which microorganisms mediate the anaerobic oxidation of C_2_–C_4_ alkanes, as many studies have indicated that the degradation of these aliphatic hydrocarbons may be linked to global biogeochemical cycles (Lorenson et al., 2002; Formolo et al., 2004; Sassen et al., 2004; Milkov, 2005; Bowles et al., 2011; Quistad and Valentine, 2011).

Recently, SRB from hydrocarbon seep sediments of the Gulf of Mexico and Guaymas Basin – both of which are environments rich in short-chain alkanes – were documented to oxidize short-chain alkanes to CO_2_ anaerobically (Kniemeyer et al., 2007). Different temperature regimens (12, 28, and 60°C) along with multiple substrates were tested and a pure culture (deemed BuS5) was isolated from mesophilic enrichments with C_3_ or C_4_ as the sole exogenous carbon source. Through comparative sequence analysis, strain BuS5 was determined to cluster with the metabolically diverse *Desulfosarcina/Desulfococcus* (DSS) cluster, which also contains the SRB found in consortia with anaerobic methanotrophs (ANME) in seep sediments. Enrichments from a terrestrial, low temperature sulfidic hydrocarbon seep corroborated the biodegradation mechanism of complete C_3_ oxidation to CO_2_ with most bacterial phylotypes surveyed belonging to the *Deltaproteobacteria*, particularly within the family *Desulfobacteraceae* (Savage et al., 2010). Cold adapted C_3_ and C_4_, sulfate-reducing cultures have also been obtained from Gulf of Mexico and Hydrate Ridge sediments with maximum rates of SR between 16 and 20°C and dominant phylotypes allied to the DSS cluster including BuS5 (Jaekel et al., 2012). In the study by Kniemeyer et al. (2007) C_4_ alkane degradation linked to sulfate reduction (SR) was not quantified at thermophilic temperatures, buta Guaymas Basin sediment enrichment with C_3_ at 60°C was dominated by Gram positive bacteria most closely allied to the *Desulfotomaculum*. Moreover, there was no evidence for C_2_degradation in mesophilic (28°C) or thermophilic (60°C) enrichments or C_2_-linked SR (albeit, there was very slow C_2_-dependent SR in Gulf of Mexico enrichments at 12°C after >200 days).

The Middle Valley (MV) hydrothermal vent field – located on the northern Juan de Fuca Ridge – is an ideal environment for investigating mesophilic to thermophilic anaerobic oxidation of C_2_–C_4_ alkanes, given the elevated temperatures and dissolved hydrocarbon species characteristic of these sediments (Goodfellow and Blaise, 1988; Davis and Fisher, 1994; Cruse and Seewald, 2006). Deep sea hydrothermal vents are complex and dynamic habitats characterized by steep thermal and chemical gradients, a diverse array of carbon and energy sources, and high concentrations of dissolved volatiles (Butterfield et al., 1990, 1994; Von Damm et al., 1995). In the MV system, hydrothermal vent fluids interact with overlying sediments and the thermal alteration of sedimentary organic matter results in the production and/or release of a number of carbon sources, including short-chain alkanes (Cruse and Seewald, 2006; Cruse et al., 2008; Cruse and Seewald, 2010). These hydrothermally influenced sediments also contain high concentrations of reduced compounds, such as H_2_ and hydrogen sulfide (H_2_S; Ames et al., 1993; Rushdl and Simonelt, 2002), and metals and metal sulfides at various reduced and oxidized states (Goodfellow and Blaise, 1988; Ames et al., 1993; Wankel et al., 2012). In contrast to the extremely organic-rich sediments of other sedimented hydrothermal systems, e.g., the Guaymas Basin hydrothermal vent fields in the Gulf of California (% OC = 2–4), MV represents a system that is more typical of mid-ocean ridge hydrothermal vents worldwide (% OC = 0.3–0.5; Wankel et al., 2012). Such environments could support the coupling of C_1_–C_4_ alkane degradation to SR in addition to alternative electron acceptors, such as metal oxides, particularly when the organic carbon load and associated SR rates are low (Wankel et al., 2012).

We studied the anaerobic oxidation of C_1_–C_4_ alkanes in metalliferous, organic-poor MV hydrothermal sediments across environmentally relevant temperature gradients. This biogeochemical investigation aimed to determine: (i) the temperature range over which hydrothermal sediment communities oxidize C_1_–C_4_ alkanes, (ii) the degree to which the anaerobic oxidation of these alkanes is coupled to SR, and (iii) the putative microbial phylotypes mediating C_1_–C_4_ alkane oxidation. To address these aims, a series of incubations were conducted using slurries of sediments collected from the MV system. These anaerobic batch reactors enabled the quantification and direct comparison of C_1_–C_4_ alkane oxidation and SR rates in a closed system across a broad range of discrete temperatures (25, 55, and 75°C). Archaeal and bacterial community dynamics were investigated via pyrotaq sequencing in select batch reactor sediments that exhibited the greatest alkane oxidation activity over the incubation time course. The overall objective of this study was to advance our understanding of the nature and extent of the anaerobic oxidation of short-chain alkanes in hydrothermal systems and to ascertain the potential influence of these processes on other biogeochemical cycles. The data presented herein shed light on the relative contribution of the anaerobic oxidation of C_2_–C_4_ alkanes at different temperature regimes, the potential influence on AOM and the sulfur cycle, and the phylotypes most likely allied to the observed metabolisms.

## MATERIALS AND METHODS

### STUDY SITE AND SAMPLE COLLECTION

Sediments were collected during an expedition with the *DSV Alvin* and *R/V Atlantis* in July 2010 from the Chowder Hill hydrothermal vent field in MV (48°27.44 N, 128°42.51W) at 2413 m depth. Intact sediment cores were recovered with polyvinylchloride core sleeves (20–30 cm height, 6.35 cm ID, 0.32 cm sleeve thickness). Sediment sampling sites were selected based on *in situ* temperature depth profiles collected with *DSV Alvin*, the presence of chemoautotrophic microbial mats atop the sediments, and shimmering water from the diffuse flow sediments. At all sites, sediment temperature profiles were collected using the RTD probe, while dissolved alkanes and other gases were quantified using an *in situ* mass spectrometer (or ISMS; data not shown; Wankel et al., 2011). Pushcores were collected from areas where sediments temperatures ranged from 5–55°C in the upper 15 cm and 57–75°C at 30 cm sediment depth. Upon retrieval, cores were sealed and refrigerated for transport to the laboratory. Upon return to the lab, the overlying water in the sediment cores was replaced weekly with fresh, filter-sterilized anoxic seawater prior to initiation of the experiments.

### ANAEROBIC BATCH REACTORS WITH C_1_–C_4_ ALKANES

In an anaerobic chamber (Coy Laboratory Products), 50 ml of homogenized whole core sediment and 50 ml of sterile, anaerobic artificial “diffuse vent fluid” were aliquoted into 200 ml glass autoclaved serum vials for each treatment. The artificial vent fluid was modified from Widdel and Bak (1992) to include 1 mM Na_2_S to ensure that sediments remained at reducing conditions, 50 mM ${Na}_{2}{SO}_{4}^{2-}$ to reduce the possibility of sulfate limitation, and the pH adjusted to 6 to mimic the diffuse vent fluids. For each incubation temperature, the headspace was pressurized to slightly above 1 atm with the respective alkane (C_1_–C_4_) or nitrogen (N_2_) gas in duplicate batch reactors to avoid alkane limitation in the aqueous phase during the incubation time series. The reactors were incubated at temperatures reflecting the sea water-sediment interface (25°C), the mid-depth average temperature (55°C), and the highest temperatures measured at the deepest depth (75°C). Flasks were shaken daily to ensure homogeneity in the slurry.

### GEOCHEMICAL MEASUREMENTS

Concentrations of the dissolved C_1_, C_2_, C_3_, and C_4_ alkanes were determined after allowing the incubations to reach room temperature and by vigorously shaking samples to transfer gas from the anaerobic seawater media to the batch reactor headspace. Then, a 0.5 ml sample of the headspace was injected into a gas chromatograph equipped with a flame ionization detector (Hewlett Packard 5890 Series II) and a packed column (RestekRt-XL) to quantify all alkanes. Injections of chemically pure alkanes (Airgas East, >99% purity) were used to generate standard curves.

Sulfate reduction rates were determined by quantifying changes in sulfate and sulfide concentrations via ion chromatography and colorimetric assays, respectively (Cline, 1969; Joye et al., 2004). After shaking and allowing the sediment to settle, a 1 ml fluid sub-sample was collected with a syringe from each reactor, filter-sterilized (0.2 μm) and transferred into a vial, preserved with 10 μl HNO_3_, and stored at 7°C until analysis. Concentrations of sulfate were determined using a Dionex ion chromatography system (Dionex Corp. Sunnyvale, CA, USA) at the University of Georgia, and NaBr, a conservation tracer in the batch reactors, was measured simultaneously. A 1 ml headspace sub-sample was collected and mixed with an equal volume of 20% zinc acetate to quantify gaseous H_2_S. Concentrations of H_2_S were then determined colorimetrically as per Cline (1969). The reported values were corrected for HS^-^dissolved in the aqueous phase and reflect both sulfide species in the serum vial headspace and sediment slurry.

### DNA EXTRACTION, MASSIVELY PARALLEL SEQUENCING, AND PHYLOGENETIC ANALYSIS

At the conclusion of each incubation, sediments were sub-sampled in an anaerobic chamber, and ~15 g of sediment slurry from each batch reactor was transferred directly into a 15 ml cryovial, flash frozen in liquid nitrogen and stored at -80°C until further molecular analyses. A time zero T_0_ sub-sample was collected at the start of the incubations to represent the initial community after homogenization, but prior to inoculation of the batch reactors. Total genomic DNA was extracted using phenol-chloroform (Barns et al., 1994; Dojka et al., 1998; Elshahed et al., 2004) modified to prevent nucleic acid loss and eliminate potential inhibitors of downstream PCR (as described in Webster et al. (2003)). Briefly, 0.5 g of sediment per batch reactor was washed with 5% HCl and then DNA was extracted with addition of 200 μg of poly adenylic acid (poly A) during the lysis step followed by incubation with lysozyme and proteinase K, multiple freeze-thaw cycles with 5% SDS, addition of hot phenol, extraction with phenol-chloroform, and elution in 50 μl TE buffer (10 mM Tris hydrochloride, 1 mM EDTA, pH 8.0). The concentration of extracts was determined using the Quant-iT^TM^ dsDNA high sensitivity Assay (Invitrogen, Carlsbad, CA, USA).

DNA extracted from the 55°C incubations, which represented the highest rates of activity, was subjected to massively parallel sequencing of the 16S ribosomal RNA (rRNA) gene using the primer pairs 27F/519R and 340F/806R for the bacterial V1 – V3 and archaeal V3 – V4 regions, respectively (Dowd et al., 2008; Acosta-Martínez et al., 2010). All pyrosequence data were submitted to the NCBI Sequence Read Archive under accession number SRA066151. The resulting reads were checked for sequence quality, trimmed, filtered, and analyzed in the software MOTHUR (Version 1.28.0; Schloss et al., 2009). Sequences were first filtered by the presence of sequence ambiguities, long homopolymers, and quality scores. The PyroNoise algorithm was then implemented in MOTHUR (i.e., shhh.flows) to remove sequences likely generated by pyrosequencing error (Quince et al., 2009). After selection of unique sequences, chimeras were identified and removed using UCHIME (http://www.drive5.com/uchime/). The resulting archaeal and bacterial reads were then aligned to the SILVA SEED Bacterial and Archaeal databases, containing 14,956 and 2,297 sequences, respectively.

For sequence classification, bootstrap values were set to nodes that had >80% support in a bootstrap analysis of 100 replicates, and operational taxonomic units (OTUs) were defined as sequences sharing 97% nucleotide sequence identity for further community analyses. A phylogenetic tree of representative *Deltaproteobacteria *(50 unique sequences selected in Mothur, i.e., sub.sample) was then generated with FastTree 2.0.0 (Price et al., 2010) using minimum-evolution subtree-pruning-regrafting and maximum-likelihood nearest-neighbor interchanges. Local support values shown are based on the Shimodaira-Hasegawa (SH) test with 1,000 resamples. Only values >80% are shown on the branches as black circles. The tree was rooted to the 16S rRNA sequence of *Archaeoglobus profundus *DSM 5631 (NR_074522).

## RESULTS

### C_1_–C_4_ ALKANE OXIDATION AS A FUNCTION OF TEMPERATURE IN BATCH REACTORS

**FIGURE 1:**
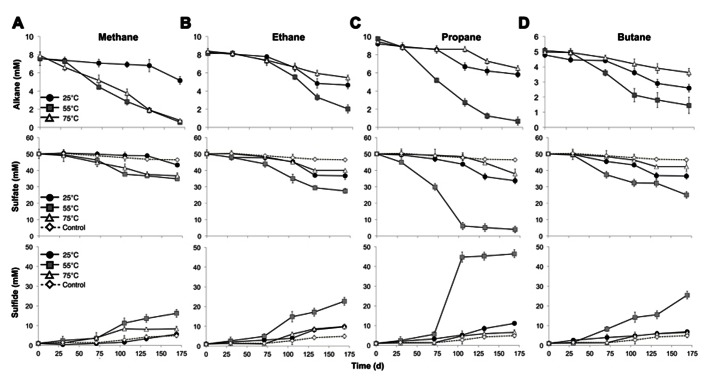
**Time course of the anaerobic oxidation of short-chain alkanes (top), consumption of sulfate (middle), and production of sulfide (bottom) in anaerobic batch reactors of Middle Valley hydrothermal sediments incubated with methane (A), ethane (B), propane (C), and butane (D) at three discrete temperatures (25, 55, and 75°C)**. Each time point represents the average of duplicate reactors with bars indicating the data range. For sulfate and sulfide measurements, the control in each panel represents the average sulfate consumption and sulfide production in the batch reactors with nitrogen for each incubation temperature as described in the text.

Batch reactor incubations were conducted using MV sediment slurries with one alkane gas (C_1_, C_2_, C_3_, or C_4_) as the sole exogenous hydrocarbon, and incubated in the laboratory at 25, 55, and 75°C to reflect the range of temperatures measured *in situ*. Temperature affected the time required to detect alkane consumption, the percent of available substrate consumed, and the absolute rates of the anaerobic oxidation of C_1_–C_4_. In batch reactors at 55°C, alkane consumption, defined as 10% of pool consumption, was evident after 71 days of incubation (**Figure 1**, top). In contrast, alkane consumption was detectable in 25°C batch reactors after 105 days for C_1_–C_4_. In 75 °C batch reactors, substantial C_2_–C_4_ consumption was apparent after 105 days; however, C_1_ consumption was evident after a much shorter time period (30 days) at 75°C.

Examining the fraction of available alkane consumed during the entire experiment (169 days), the greatest total consumption of C_1_–C_4_ occurred in the 55°C batch reactors (~93, 75, 93, and 77% of C_1_, C_2_, C_3_, and C_4_, respectively). In addition, C_1_ was nearly depleted in the 75°C batch reactors by the end of the time series (with >90% substrate consumed). With the exception of C_1_ at 75°C, less than half of the available short-chain alkane pool was consumed during the incubation time course in 25 and 75°C batch reactors (32, 44, 37, and 46% of C_1_, C_2_, C_3_, and C_4_ at 25°C, respectively, and 35, 31, and 27% of C_2_, C_3_, and C_4_ at 75°C, respectively).

Absolute rate measurements of the batch reactor sediments revealed that maximum C_1_–C_4_ oxidation occurred at 55°C (~42, 36, 54, and 23 nmol cm^-3^day^-1^ for C_1_, C_2_, C_3_, and C_4_, respectively, *n *= 2; **Table 1**). Substantially lower rates of the anaerobic oxidation of C_2_–C_4_were observed in all 25 and 75°C batch reactors (~21, 16, and 8 nmolcm^-3^day^-1^ for C_2_, C_3_, and C_4_ at 25°C, respectively, *n *= 2, and ~17, 17, and 8 nmolcm^-3^day^-1^ for C_2_, C_3_, and C_4_ at 75°C, respectively, *n *= 2). In contrast to the other short-chain alkanes, maximal rates of AOM were also observed at 75°C (~42 nmolcm^-3^day^-1^, *n *= 2), while rates decreased to less than half of these AOM maxima at 25°C (14 nmolcm^-3^day^-1^, *n *= 2).

**Table 1:** Volume-specific rate measurements of the anaerobic oxidation of methane, ethane, propane, and butane and sulfate reduction in batch reactors incubated at 25, 55, and 75°C.

	Anaerobic oxidation nmol cm^–3^ day^–1^	Sulfate reduction nmol cm^–3^ day^–1^
Methane – 25°C	14.33 ± 2.88	15.01 ± 2.31
Methane – 55°C	41.45 ± 1.17	55.83 ± 4.91
Methane – 75°C	42.22 ± 1.91	68.03 ± 5.01
Ethane – 25°C	21.39 ± 4.77	53.61 ± 6.53
Ethane – 55°C	36.03 ± 4.46	99.30 ± 8.48
Ethane – 75°C	17.22 ± 3.59	47.94 ± 3.65
Propane – 25°C	19.93 ± 2.81	71.96 ± 7.25
Propane – 55°C	53.66 ± 2.52	238.36 ± 10.77
Propane – 75°C	17.26 ± 1.26	60.37 ± 6.18
Butane – 25°C	12.84 ± 2.81	55.27 ± 8.68
Butane – 55°C	23.07 ± 5.13	113.46 ± 15.37
Butane – 75°C	8.01 ± 1.13	34.22 ± 2.39

*Rates were determined from the consumption of alkanes and sulfate over the incubation time course. Each point represents the average of duplicate reactors with the standard error. To account for background sulfate reduction (due to autochthonous carbon, etc.), the rates measured in the alkane treatments have been corrected via subtraction of those measured in the control (nitrogen) batch reactors.*

### SULFATE REDUCTION COUPLED TO C_1_–C_4_ ALKANE OXIDATION ACROSS TEMPERATURE REGIMES

In addition to a dependence on short-chain alkane length, temperature constrained SR in the anaerobic batch reactors, influencing quantified changes in porewater sulfate and total sulfide. Decreases in sulfate concentration were observed in all batch reactors across time and temperature regimes, consistent with trends for the anaerobic oxidation of C_1_–C_4_ alkanes. Analogous to alkane consumption dynamics, sulfate consumption was appreciable (defined as >10% substrate consumption) after 71 days of incubation in C_1_–C_4_ batch reactors at 55°C (**Figure 1**, middle). In contrast, there was a lag of ~105 days in C_2_, C_3_, and C_4_ batch reactors prior to substantial sulfate consumption at both the lowest (25°C) and highest (75°C) incubation temperature. Over the span of the incubation time series (169 days), the greatest reduction in sulfate concentration was at 55°C (~30, 45, 92, and 49% of total sulfate consumed in the C_1_, C_2_, C_3_, and C_4_ reactors, respectively). Sulfate consumption was also observed in the N_2_-control batch reactors, albeit to a much smaller extent (~8, 11, and 2% at 25, 55, and 75°C, respectively). SR was also assessed by quantifying the production of gaseous and dissolved sulfide in the batch incubations (**Figure 1**, bottom). In all reactors, sulfide concentrations at the end of each incubation time period accounted for greater than 90% of the initial total sulfate plus sulfide concentration; therefore, these mass balance estimates were within 10% of the total sulfur species observed initially.

Concomitant with the anaerobic oxidation of C_2_–C_4_ rates, maximum SR rates were observed at 55°C for the non-methane short-chain alkanes (~99, 238, and 113 nmolcm^-3^day^-1^ for C_2_, C_3_, and C_4_, respectively, *n *= 2) (**Table 1**). However, maximum SR rates associated with AOM occurred at 75°C (~68 nmolcm^-3^day^-1^), with lower rates at 55°C (~55 nmolcm^-3^day^-1^, *n *= 2) and even more modest rates at 25°C (~15 nmolcm^-3^day^-1^, *n *= 2). In comparison to maximal SR rates at 55°C, SR rates linked to C_2_–C_4_ oxidation were lower at both 25 and 75°C (~54, 72, and 55 nmolcm^-3^day^-1^ for C_2_, C_3_, and C_4_ at 25°C, respectively, *n *= 2, and ~48, 60, and 34 nmolcm^-3^day^-1^ for C_2_, C_3_, and C_4_ at 75°C, respectively, *n *= 2).

The observed ratio (mol/mol) of C_1_–C_4_ oxidation to SR in the batch reactors was then compared to the predicted stoichiometric ratio assuming the sulfate-dependent complete oxidation of C_1_–C_4_ alkanes to CO_2_ (from Kniemeyer et al., 2007). These ratios are corrected for the consumption of sulfate in the control (N_2_) batch reactors as an estimate for SR linked to non-alkane organic carbon donors present in the sediment. The ratio of mol alkane consumed per mol sulfate reduced was 1.42, 1.11, and 0.93 mmol of C_1_ mmol^-1^ sulfate; 0.59, 0.54, and 0.54 mmol of C_2_ mmol^-1^ sulfate; 0.42, 0.34, and 0.43 mmol of C_3_ mmol^-1^ sulfate; and 0.35, 0.31, and 0.35 mmol of C_4_ mmol^-1^ sulfate at 25, 55, and 75°C, respectively (**Table 2**). These ratios closely mirror the predicted stoichiometric ratios of 1, 0.5, 0.4, and 0.3 for C_1_–C_4_, respectively.

**Table 2:** The predicted and calculated stoichiometric ratios for the anaerobic oxidation of methane, ethane, propane, and butane coupled to the reduction of sulfate to sulfide.

	Stoichiometric Ratio (mol/mol)	Observed Ratio (mol/mol)
Methane – 25°C	1	1.42
Methane – 55°C	1	1.11
Methane – 75°C	1	0.93
Ethane – 25°C	0.5	0.59
Ethane – 55°C	0.5	0.54
Ethane – 75°C	0.5	0.54
Propane – 25°C	0.4	0.42
Propane – 55°C	0.4	0.34
Propane – 75°C	0.4	0.43
Butane – 25°C	0.31	0.35
Butane – 55°C	0.31	0.31
Butane – 75°C	0.31	0.35

*From closed system batch reactors, the mol alkane lost was calculated per mol sulfate reduced at 25, 55, and 75°C, respectively. To account for background sulfate reduction (due to autochthonous carbon, etc.), ratios have been corrected via subtraction of those measured in the control (nitrogen) batch reactors.*

### PHYLOGENETIC DIVERSITY AND DISTRIBUTION IN SEDIMENTS FROM BATCH C_1_–C_4_ REACTORS

**FIGURE 2:**
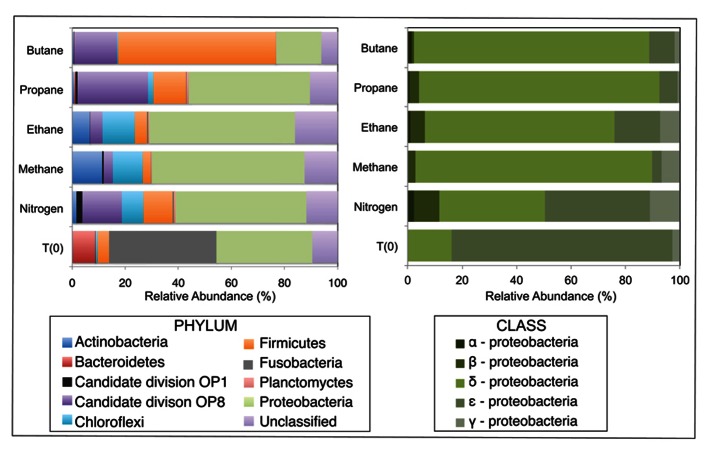
**Relative abundance (percentage) of bacteria determined from massively parallel sequencing of DNA recovered from anaerobic batch reactor sediments incubated with methane, ethane, propane, butane, and nitrogen at 55°C and pre-incubation (T_0_) sediments**. Left and right side panels show the taxonomic breakdown of sequences at the phylum and class level, respectively. Legend indicates operational taxonomic units (OTUs), defined as sequences sharing 97% nucleotide sequence identity.

After sequence processing and denoising as previously described, a total of 5783, 6562, 5307, 6985, and 8796 bacterial sequences were analyzed from sediments incubated with N_2_, C_1_, C_2_, C_3_, and C_4_ alkane, respectively, and 7965 bacterial sequences from the T_0_ sediment. There were substantial shifts at the phyla level between the communities incubated with different alkane substrates in comparison to the control batch reactor and T_0_ sediment community (**Figure 2**). From the initial sediment community, sequences allied to the *Bacteroidetes* and *Fusobacteria* decreased from ~9 and 40% of T_0_ sequences respectively, to less than 0.5% of sequences in all batch reactor libraries. In turn, sequences allied to the *Proteobacteria*, *Firmicutes*, Candidate Division OP8, *Chloroflexi*, and *Actinobacteria* increased in batch reactor libraries compared to T_0_ sequences. Notably, the *Proteobacteria, *which comprised ~36% of T_0_ sequences, increased in representation in the N_2_, C_1_, C_2_, and C_3_ sequence libraries (~49, 58, 41, and 46%, respectively). The *Firmicutes *also increased substantially from the T_0_ composition (~4%) in N_2_, C_2_, C_3_, and C_4_ sequences (~11, 12, 12, and 59%, respectively).

Among the *Proteobacterial* sequences allied to known sulfate-reducing *Deltaproteobacteria*, there was a substantial increase from T_0_ sequences (~15%) in the N_2_, C_1_, C_2_, C_3_, and C_4_ sequence libraries (~39, 87, 70, 88, and 86%, respectively). Concurrently, there was a substantial decrease in the representation of *Epsilonproteobacteria* in the N_2_, C_1_, C_2_, C_3_, and C_4_ sequence libraries (~39, 3, 17, 7, and 9%, respectively). Within the putative sulfate-reducing phylotypes, the C_1_ library was comprised primarily (>92%) of sequences allied to *Desulfobulbus*, as shown in a previous study of MV sediment communities associated with AOM (Wankel et al., 2012). Analysis of 16S rRNA gene libraries revealed that a distinct lineage of SRB are the predominant *Deltaproteobacterial* phylotypes in the C_2_–C_4_ reactor communities, comprising ~93, 91, and 95% of C_2_, C_3_, and C_4_ sequences, respectively (**Figure 4**). The most closely related phylotypes (93–99% nucleotide sequence identity) were previously recovered in two 16S rRNA-based surveys of sulfate-reducing anaerobic enrichments of Guaymas Basin sediments with C_4_ at 60°C (Butane60-GuB, accession no. EF077228) and with C_1_ at 37°C (Guaymas_Bac9 clone, accession no. FR682643; Kniemeyer et al., 2007; Kellermann et al., 2012).

**FIGURE 3:**
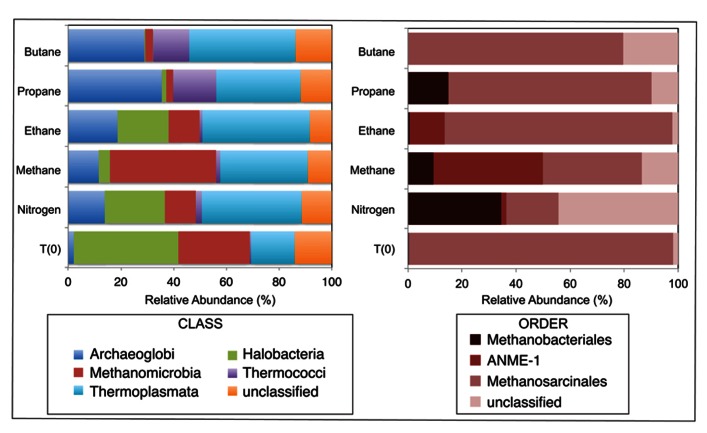
**Relative abundance (percentage) of archaea determined from massively parallel sequencing of DNA recovered from anaerobic batch reactor sediments incubated with methane, ethane, propane, butane, and nitrogen at 55°C and pre-incubation (T_0_) sediments**. Left and right side panels show the taxonomic breakdown of sequences at the class and order level, respectively. Legend indicates operational taxonomic units (OTUs), defined as sequences sharing 97% nucleotide sequence identity.

A total of 1290, 1724, 1540, 1916, 1780, and 2846 Euryarchaeotal sequences were further analyzed from the N_2_, C_1_, C_2_, C_3_, and C_4_ batch reactors and T_0_ sediments, respectively (**Figure 3**). There were notable shifts in the sequences allied to the predominant Euryarchaeotal phyla – *Archaeoglobi*, *Halobacteria*, *Methanomicrobia*, *Thermococci*, and *Thermoplasmata* – from the initial sediment community and across the different alkane batch incubations. Over 40% of sequences were allied to the *Halobacteria* in T_0_ sediments, decreasing to comprise <0.5–29% of batch reactor sequences. In contrast, *Archaeoglobi *sequences increased from ~2% of T_0_ sequences to ~14, 12, 19, 36, and 29% of N_2_, C_1_, C_2_, C_3_, and C_4_ sequences, respectively. Other trends in Euryarchaeotal community structure included an increase in *Methanomicrobia* from 27% of T_0_ sequences to 40% of C_1_ sequences.

Within the *Methanomicrobia*, there were also substantial changes in sequences allied to known methanogens and methane-oxidizing phylotypes. *Methanosarcinales *comprised >97% of T_0_ sequences and ~19, 36, 84, 75, and 80% of N_2_, C_1_, C_2_, C_3_, and C_4_ sequences, respectively. In contrast, *Methanobacteriales* increased from <0.5% of T_0_ sequences to ~35, 9, and 26% of N_2_, C_1_, and C_3_ sequences (there was no substantial increase in C_2_ or C_4_ sequences). For the putative methane-oxidizing communities, over 40 and 12% of C_1_ and C_2_ sequences were allied to ANME-1 ribotypes.

### DISCUSSION

The microbial degradation of short-chain alkanes under oxic conditions and the anaerobic oxidation of methane and other heavier hydrocarbons have been extensively studied in diverse terrestrial and marine environments. Despite studies indicating short-chain alkane degradation in anoxic deep sea sediments (Sassen et al., 2004; Mastalerz et al., 2009; Quistad and Valentine, 2011) and the abundance of short-chain alkanes in hydrocarbon-rich ecosystems (Milkov, 2005; Cruse and Seewald, 2006), relatively little is known about the biogeochemical importance of these processes or the diversity of anaerobic short-chain alkane degrading microorganisms in marine hydrothermal sediments. The data here provide a deeper glimpse into the anaerobic oxidation of C_2_–C_4_ in metalliferous hydrothermal sediments and reveal that rates of the anaerobic oxidation of C_2_–C_4_ alkanes in hydrothermal vent sediment are heavily influenced by temperature and coupled to SR, though the rates presented herein are derived from conditions not likely to be present *in situ*, and as such care should be taken when extrapolating these rates to natural processes. In batch reactor sediments that exhibited the most substantial activity, changes in the representation of phylotypes in libraries generated via high throughput sequencing implicate *Deltaproteobacteria* in C_2_–C_4_ alkane degradation, and shifts in microbial community composition indicate that other members of the community respond to the presence of short-chain alkanes (though the mechanisms underlying this response remain unknown).

These data revealed a preferential consumption of C_2_–C_4_ at 55°C, suggesting that the active alkane degraders in these hydrothermal vent sediments are thermophilic. Furthermore, these *ex situ* calculated rates for the anaerobic oxidation of C_2_–C_4_ were in the same range (nmolcm^-3^day^-1^) as the recently reported anaerobic oxidation of C_3_ in marine hydrocarbon seep sediments and as AOM rates measured in organic-rich coastal sediments at the sulfate-methane transition zone (Alperin et al., 1988; Hoehler et al., 1994; Girguis et al., 2003; Wegener et al., 2008; Quistad and Valentine, 2011). Based on lag time and total alkane degraded over time, C_3_ appeared to be the preferred substrate in the 55°C incubations, followed by C_1_, C_4_, and C_2_, respectively. Similar trends in the biodegradation of short-chain alkanes have been found in stable isotopic studies of hydrocarbon reservoirs at temperatures below 60°C with a preference for C_3_ followed by C_4_ and then C_2_ (Boreham et al., 2001; Wenger et al., 2002; Larter et al., 2005).

Various physicochemical and biotic parameters may impact the degree of C_2_–C_4_ consumption in *ex situ* studies and in the natural environment. Notably, the gaseous alkanes were maintained at above saturation conditions for the liquid phase of the batch incubations until the end of the time series to ensure substrate availability (dissolved concentrations of ~1.42, 1.89, 0.91, and 1.05 mM for C_1_, C_2_, C_3_, and C_4_, respectively). Under elevated hydrostatic pressure in the deep sea, hydrothermal vent fluids at MV reach C_1_ concentrations of ~20 mM, while the other short-chain alkanes are an order of magnitude lower (~220, 55, and 6 μM for C_2_, C_3_, and C_4_, respectively; Cruse and Seewald, 2006). Although C_1_ is most likely more abundant than C_3_ in MV hydrothermal sediments, the *in situ *rates of C_3_ degradation may be appreciable due to the inherent reactivity of secondary C-H bonds (Schink and Friedrich, 1994; Rabus et al., 2001; Van Hamme et al., 2003).

Our results also suggest that, at the highest incubation temperatures, AOM in MV sediments occurs at higher rates than the anaerobic oxidation of C_2_–C_4_ alkanes. In the higher temperature (75°C) incubations, C_1_ consumption was evident after 30 days and reached near deplete concentrations (90% substrate consumed), while there was a much longer lag period until C_2_–C_4_ degradation (105 days) and much less of the substrates were consumed by the completion of the time series (27–35%; **Figure 1**, top). In contrast, a greater proportion of C_2_ and C_4_ (44 and 46%, respectively) were consumed than C_1_ and C_3_ (32 and 37%, respectively) in the lower temperature incubations (25°C). The increased AOM activity at the higher end of the temperature range in MV sediments is consistent with our previous observations of AOM in these metalliferous sediments (Wankel et al., 2012), and is also consistent with the growth temperatures of archaeal communities (such as ANME phylotypes) from hydrothermal vents, which indicate that many archaea live at their maximum growth temperature *in situ* (Kimura et al., 2013). Another line of evidence for thermophilic AOM was also provided in a recent 16S rRNA based-study identifying a putatively high temperature-adapted ANME subgroup in both hydrothermal sediments from Guaymas Basin and diffuse vent fluids from Axial Volcano and the Endeavor Segment of Juan de Fuca Ridge (Merkel et al., 2013).

Notably, the anaerobic oxidation of C_1_–C_4_ was coupled to SR across temperature gradients in MV sediment batch reactors. Sulfate loss (~2–6 mM) was also observed over the time series in alkane-free control batch reactors (**Figure 1**, middle). In comparison to SR linked to the oxidation of short-chain alkanes, this modest sulfate consumption relates to the oxidation of endogenous substrates, particularly organic carbon, by the sediment communities (Gieg et al., 1999). The sediment organic carbon pool of MV sediments (% OC = ~0.5 in this study) is low in comparison to the high amounts of organic matter that characterize other deep sea environments with known short-chain alkane degraders, such as the organic-rich Guaymas Basin hydrothermal sediments (Jorgensen et al., 1992; Kniemeyer et al., 2007). The observed SR rates in C_1_–C_4_ batch reactors of MV sediments demonstrate the potential for organic carbon-poor, high temperature mid-ocean ridge systems to support the anaerobic oxidation of short-chain alkanes coupled to SR.

Our results further indicate that short-chain alkane degradation linked to SR might considerably influence sulfate cycling at these sedimented hydrothermal vents. In accordance with the observed stoichiometries, SR coupled to the anaerobic oxidation of C_2_, C_3_, and C_4_ proceeded at a faster rate than AOM at mesophilic and thermophilic temperatures (25 and 55°C, respectively). However, the SR rates in anaerobic batch reactors were observed under sulfate-replete conditions, while the sulfate pool *in situ* depends on the downward advection of seawater and the activity of sulfide-oxidizing microbial communities (Bowles et al., 2011). Sulfate availability will become limiting at greater sediment depths from the seawater surface. Therefore, the C_2_, C_3_, and C_4_-degrading, sulfate-reducing microbial communities likely compete for available sulfate and might indirectly limit AOM in the temperature range from ~25–55°C. As previously discussed, the anaerobic oxidation of these aliphatic hydrocarbons coupled to the reduction of sulfate to sulfide yields greater energy per unit substrate than AOM. Such processes could constrain methane release from the deep-sea with a critical impact on the global carbon cycle and climate. Furthermore, if AOM activity peaks at greater sediment depths and higher temperatures *in situ* as predicted by rate measurements, then sulfate will most likely have been depleted in these sediment horizons. Sulfate limitation may thus result in the coupling of AOM to alternative electron acceptors (i.e., iron oxides), as indicated in previous studies of MV high temperature sediment incubations (Wankel et al., 2012).

**FIGURE 4:**
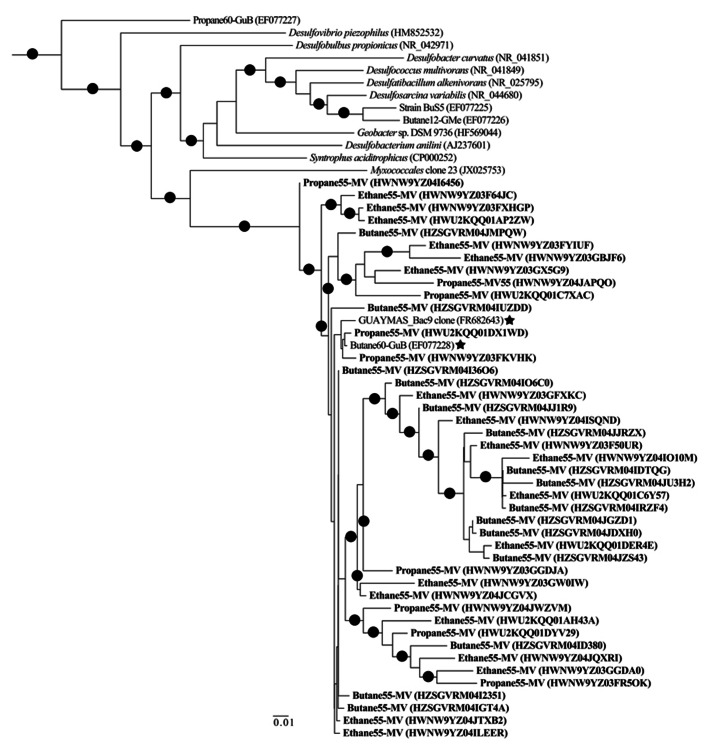
**Maximum-likelihood phylogenetic tree illustrating the relationships of selected 16S rRNA *Deltaproteobacterial* sequences recovered from Middle Valley sediments to *Deltaproteobacterial *sequences and uncultured environmental phylotypes from NCBI non-redundant database**. Representative sequences from Middle Valley sediments incubated in batch reactors at 55°C with ethane (Ethane55-MV), propane (Propane55-MV), and butane (Butane55-MV) in bold. The most closely related environmental phylotypes from Guaymas Basin sediment sequences have been marked (**⋆**). The tree was rooted to *Archaeoglobus profundus *DSM 5631(NR_074522). Scale = 0.01 substitutions per site.

Comparison of bacterial communities in batch reactor sediments with maximum rates of C_1_–C_4_ degradation, via massively parallel pyrosequencing, suggests that members of the sulfate-reducing *Deltaproteobacteria* mediate the anaerobic oxidation of short-chain alkanes in MV hydrothermal vent sediments (**Figure 2**). As these sequence data are based on PCR amplification of 16S rRNA genes and are semi-quantitative, an order of magnitude difference in phyla should represent shifts in community composition. Within the *Proteobacteria*, there was a substantial increase of *Deltaproteobacteria *in C_1_–C_4_ sequences compared to the initial T_0_ bacterial composition dominated by *Epsilonproteobacteria*. Phylogenetic analyses revealed a novel subgroup of SRB that comprised >90% of these *Deltaproteobacteria *in C_2_–C_4_ batch reactor sequences (**Figure 4**). This lineage of *Deltaproteobacteria *is most closely related to C_4_-degrading SRB from Guaymas Basin, and therefore, may be a thermophilic short-chain alkane degrader group (Kniemeyer et al., 2007). Intriguingly, the predominant phylum in C_4_ batch reactor sequences is the *Firmicutes*, which contains sulfate-reducing members of previous enrichments with C_3_ and C_4_ (Kniemeyer et al., 2007; Savage et al., 2010). However, the majority of *Firmicutes* sequences were most closely related (98–99%) to uncultured *Bacillus* clones from hydrocarbon-contaminated soils (Wang et al., 2011). The greater proportion of this uncharacterized *Bacillus* group in comparison to SRB may have also affected the lower rates of the anaerobic oxidation of C_4_ in comparison to C_2_ or C_3_ in batch incubations. Future studies should determine if this putative thermophilic short-chain alkane degrader group of SRB is widespread in other hydrothermally influenced environments.

Amongst the batch reactor sediment communities, shifts in archaeal phylogenetic composition were also revealed via 16S rRNA pyrosequencing. There was a substantial increase of *Methanomicrobia* sequences in C_1_-incubated sediments compared to the initial community and C_2_–C_4_ batch reactor sediments, with an order of magnitude enrichment of ANME-1 phylotypes within the *Methanomicrobia* (**Figure 3**). The microbes known to catalyze AOM form three phylogenetically distinct Euryarchaeaota clusters (ANME-1, ANME-2, and ANME-3) that often appear to live in consortia with SRB (Hoehler et al., 1994; Boetius et al., 2000; Orphan et al., 2001). However, ANME-1 phylotypes are also found as single cells in sediments, and recent studies have shown that AOM can occur in the absence of SR and that some ANME are not directly dependent on SRB activity (Beal et al., 2009; Milucka et al., 2012; Wankel et al., 2012). There was also a notable increase in *Archaeoglobus *sequences in C_1_ – C_4_ batch reactors from the initial community composition (**Figure 3**), which contain hyperthermophilic species known to mediate SR (Shen and Buick, 2004). Based on microbial isolates and enrichments from both deep sea and terrestrial ecosystems, no evidence to date indicates that non-methane short-chain alkanes are anaerobically oxidized by microbial consortia or sulfate-reducing archaea (Kniemeyer et al., 2007; Savage et al., 2010; Jaekel et al., 2012). The data presented herein lack the resolution to conclusively address whether archaeal phylotypes directly mediate or are members of consortia that perform the anaerobic oxidation of short-chain alkanes other than AOM.

The collective results presented here shed light on the potential anaerobic metabolism of short-chain alkanes linked to SR in the hydrothermal vent sediments of MV, Juan de Fuca Ridge. Substantial oxidation of C_1_–C_4_ occurs up to 75°C. The coupling of C_2_–C_4_ with SR over the *in situ *temperature range may impact AOM and the oxidation of other hydrocarbons, as highlighted by the preferential degradation of C_3_ at 55°C. Such microbial communities may play a substantial role in carbon and sulfur cycling at hydrothermal systems on a global-scale. Future studies should expand upon other environmental conditions that may regulate the anaerobic oxidation of C_2_–C_4_ alkanes in hydrothermal sediments and should further characterize the *in situ* abundance and activity of the putative thermophilic alkane-degrader SRB lineage.

## CONTRIBUTIONS

Melissa M. Adams, Peter R. Girguis, and Arpita Bose designed the research. Melissa M. Adams, Peter R. Girguis, and Adrienne L. Hoarfrost directed the *in situ* collections and measurements. Melissa M. Adams, Adrienne L. Hoarfrost, and Arpita Bose conducted the batch reactor incubations and geochemical analyses. Samantha B. Joye performed the sulfate consumption measurements. Melissa M. Adams directed and analyzed the alkane consumption, sulfide production, and all rate calculations. Melissa M. Adams performed the molecular analyses. Melissa M. Adams and Peter R. Girguis wrote the manuscript with input from Samantha B. Joye, Arpita Bose, and Adrienne L. Hoarfrost.

## Conflict of Interest Statement

The authors declare that the research was conducted in the absence of any commercial or financial relationships that could be construed as a potential conflict of interest.
